# Effects of dietary rumen-degradable protein on growth performance, nitrogen metabolism, and rumen microbiome in dairy buffalo heifers

**DOI:** 10.3389/fvets.2026.1806578

**Published:** 2026-04-22

**Authors:** Qingfeng Tang, Dongwen Qiu, Chongli Wen, Zeming Bu, Yali Huang, Caixia Zou, Haoyi Wu, Fuchang Chen, Lin Liu, Zhonghao Li, Xiaoling Xie, Haoting Huang, Kaiwen Gan, Yiting Liu

**Affiliations:** 1 Guangxi Key Laboratory of Animal Breeding, Disease Control and Prevention, College of Animal Science and Technology, Guangxi University, Nanning, Guangxi, China; 2 Buffalo Research Institute, Chinese Academy of Agricultural Sciences, Nanning, Guangxi, China; 3 Guangxi Monitoring Institute of Feed, Nanning, Guangxi, China; 4 The Guangxi Agricultural Vocational and Technical University, Nanning, Guangxi, China

**Keywords:** growing dairy buffaloes, nitrogen utilization, rumen microbiota, rumen nitrogen supply, ruminal and serum parameters, weight gain

## Abstract

**Introduction:**

Buffaloes are globally important dairy animals, but their low feed nitrogen utilization efficiency and excessive dietary rumen-degradable protein (RDP) results in aggravated nitrogen pollution and high breeding costs. Studies on the optimal RDP levels for 7–10-month-old dairy buffalo heifers remain scarce, limiting precise nutritional management.

**Materials and methods:**

Dairy buffalo heifers (*n* = 36, 7–10-month-old, 193.39 ± 4.10 kg) were selected, and randomly assigned to six groups (*n* = 6 heifers/group, with one heifer in each replicate; Dietary RDP: 60.85–88.90 g/kg). The 73-day trial (15-day adaptation) included measurements of growth performance, nitrogen metabolism, serum indices, rumen parameters, and microbiome (16S rRNA/ITS sequencing).

**Results:**

(1) No differences in initial/final body weight or dry matter intake were found among the groups (*p* > 0.05). The low-RDP group (LP-1, 67.31 g/kg) had the highest average daily gain (0.79 kg/d) and lowest feed-to-gain ratio (7.88) (*p* < 0.05). (2) With a decrease in dietary RDP levels, intake nitrogen (IN), urinary nitrogen (UN), digested nitrogen, and UN /IN efficiency decreased (*p* < 0.05). The low-RDP group (LP-1) had the highest retention nitrogen/IN efficiency (32.31%) (*p* < 0.05). (3) The serum total protein and urea levels decreased with decreasing dietary RDP levels (lowest in LP-2: 64.52 g/L and 5.15 mmol/L, *p* < 0.05), with no differences in liver or kidney function or glucose-lipid metabolism (*p* > 0.05). (4) LP-1 had the highest rumen total volatile fatty acids, acetate, and butyrate levels (*p* < 0.05), while rumen pH and NH_3_-N decreased with RDP (*p* < 0.05). (5) Dietary RDP levels significantly altered the rumen microbial structure. *Pichia* in LP-1 was 28.81-fold and 39.68-fold higher than in HP-1 and MP-1 groups, respectively (*p* < 0.05), along with the presence of group-specific taxa.

**Discussion:**

An optimal dietary RDP level for 7–10-month-old dairy buffalo heifers was 67.31 g/kg, which improved the ADG and nitrogen utilization efficiency without compromising health, while also altering the rumen microbial structure. Therefore, when formulating diets for buffaloes, it is advisable to consider to note only meet the DCP requirements but also appropriately regulate the dietary RDP levels.

## 1 Introduction

Buffaloes are important dairy animals, supporting the livelihoods of over 2 billion people worldwide. With a global population exceeding 200 million head, buffaloes contribute more than 15% of the total annual milk production worldwide (1, 2). Recent studies have shown that buffalo milk, with its high nutritional value and unique bioactive components, can exert beneficial effects on human metabolic health and immune function (3, 4). Thus, research that focuses on improving the nutritional regulation and production efficiency of dairy buffaloes and for sustainable livestock development is warranted (5).

Low feed nitrogen utilization efficiency remains a serious bottleneck in commercial dairy buffalo farming. For instance, the nitrogen utilization efficiency is only 18–24%, with the majority excreted via feces and urine (6, 7). Maximizing nitrogen use efficiency in ruminants is most effectively achieved by avoiding diets with excessively high crude protein levels, particularly those containing excess rumen-degradable protein (RDP) (8). Dietary crude protein (CP) represents the most costly nutrient in ruminant diets, and is conventionally divided into rumen-degradable protein (RDP) and rumen-undegradable protein (RUP). The nitrogen supplied by RDP promotes the growth of rumen microorganisms, which enhances microbial activity and subsequently increases the flow of microbial nitrogen to the small intestine (9). Increasing dietary CP content leads to a corresponding increase in RDP supply; however, when RDP exceeds the nutritional requirements of rumen microorganisms, excessive ammonia (NH_3_) is produced (10). This NH_3_ is absorbed into the bloodstream, converted to urea in the liver, and ultimately excreted in the urine. Additionally, urinary urea in feces is rapidly hydrolyzed back to NH_3_, which is then released into the environment via volatilization (10–12). This inefficiency increases feed costs for milk production and exacerbates environmental challenges associated with nitrogen pollution, such as soil, water, and air contamination (13, 14).

Numerous studies have investigated the effects of dietary CP and RDP levels on nitrogen utilization and performance in ruminants. For example, increasing dietary CP from 14.1 to 20.1% improved milk yield and milk protein content in dairy cows but reduced the nitrogen use efficiency (NUE) and increased nitrogen excretion into the environment (15). In separate studies, Agle et al. (16) and Zhu et al. (17) reported that diets with low CP or low RDP enhanced the NUE, satisfied the nutritional needs of rumen microorganisms, and had no adverse effects on the growth performance of ruminants. Rastgoo et al. (18) reported that different RUP: RDP ratios (low ratio = 28:72; high ratio = 37:63) in a starter diet (20% CP on a dry matter (DM) basis, supplemented with 5% chopped wheat straw) had no effects on feed intake, average daily gain (ADG), or body weight of dairy calves. Nevertheless, calves in the high RUP: RDP group showed increased body height and hip height, suggesting that decreasing RDP intake could promote skeletal development in growing calves under the study conditions (18). A recent study on 13–15-month-old dairy buffalo heifers found that reducing dietary RDP from 80.36 g/kg DM to 72.79 g/kg DM (and CP from 15.42 to 14.03% on a DM basis) maintained the production performance of heifers while effectively improving the NUE and reducing urinary nitrogen (UN) excretion (19). Collectively, these studies confirm that an appropriate dietary RDP level is crucial for optimizing NUE, thereby reducing production costs and improving economic benefits. However, systematic research on the optimal dietary RDP content for 7–10-month-old dairy buffalo heifers—a crucial early growth stage—is lacking.

In the present work, we hypothesized that decreasing dietary CP by reducing RDP, but satisfying the digestible crude protein (DCP) requirements of 7–10-month-old dairy buffalo heifers, would not compromise their growth performance. The objective of this study was to assess the effects of reducing the dietary RDP and CP levels on the growth performance, nitrogen metabolism, serum biochemical indicators, rumen fermentation characteristics, and rumen microbial community of 7–10-month-old dairy buffalo heifers.

## 2 Materials and methods

### 2.1 Experimental animals and design

This feeding trial was conducted from June 3, 2024 to August 14, 2024, at the Buffalo Research Institute breeding farm, located in the Guangxi Zhuang Autonomous Region of Southern China (22°53′22.59″N, 108°21′51.19″E). During the experimental period, the average monthly precipitation was 390 mm, with the temperature ranging from 26 °C to 32 °C. A total of 36 dairy buffalo heifers, including Murrah, Nili-Ravi, Mediterranean hybrid buffalo, and triple-crossbred buffaloes (local Guangxi swamp buffalo × Murrah buffalo × Nili-Ravi buffalo), were selected and randomly divided into six groups (*n* = 6 heifers/group, with one heifer in each replicate), with similar body weights (193.39 ± 4.10 kg) and ages (7–10 months). The 36 dairy buffalo heifers selected for this experiment were born and raised at the Buffalo Research Institute breeding farm. All buffaloes were administered 1% ivermectin (Hebei Veyong Pharmaceutical Co., Ltd., Shijiazhuang, China) to manage and control gastrointestinal parasites. The FOM and RDP values of the main dietary components were obtained based on the effective degradability of feedstuffs acquired in the preliminary experimental results and an overview of the nutritional characteristics of the feeds utilized in this study (Table 1) (20). Six different diets were formulated according to the FOM and RDP values of the primary dietary components. These diets had various RDP values but the same FOM value (0.51 kg/kg). The diets were classified as high RDP (HP-1, RDP = 88.90 g/kg, CP = 15.55%; HP-2, RDP = 83.53 g/kg, CP = 14.68%), medium RDP (MP-1, RDP = 78.83 g/kg, CP = 13.84%; MP-2, RDP = 72.17 g/kg, CP = 12.86%), and low RDP (LP-1, RDP = 67.31 g/kg, CP = 12.02%; LP-2, RDP = 60.85 g/kg, CP = 11.02%). Based on the previous studies of Zou (21) and Ruan et al. (22), the DCP and NE_L_ were formulated separately to meet the N and energy requirements of dairy buffalo heifers. The HP-1, HP-2, MP-1, MP-2, LP-1, and LP-2 diets provided DCP balances of 268.14, 232.60, 190.16, 138.39, 104.77, and 55.69 g/d, respectively. The diets were formulated to exceed the energy requirements (5.15 kg/d of dry matter intake [DMI]) of dairy buffalo heifers with an ADG of 0.6 kg/d and a body weight of 206 kg. Table 2 presents the ingredients and chemical compositions of the diets with different RDP contents. The entire trial lasted 73 days, including a 15-day preliminary phase for feeding preparation, followed by a 58-day main period dedicated to data collection. During the final 5 days, digestion and metabolism of nitrogen (N) were evaluated by measuring the total fecal and urinary outputs from three randomly chosen buffaloes per group. From day 1 to day 73, the buffaloes were provided with fed twice daily, at 08:00 h and 16:00 h, and were allowed unrestricted access to water. Throughout the study, the buffaloes were fed ad libitum, with daily refusals maintained at approximately 5% of the feed provided to ensure unrestricted intake.

**Table 1:** Nutritional characteristics of the feedstuffs utilized in the study (DM basis,%).

Items	Corn stalk silage	Peanut vine	Corn	Soybean meal	Wheat bran
DM^a^ (%)	94.92	93.94	95.26	94.28	92.53
CP^b^ (%)	12.44	9.38	9.51	48.8	18.23
Ash (%)	9.24	9.78	1.46	7.06	6.41
OM^c^ (%)	90.76	90.22	98.54	92.94	93.59
NDF^d^ (%)	60.82	57.79	12.33	19.94	42.82
ADF^e^ (%)	33.21	42.19	2.25	7.49	11.73
FOM^f^ (kg/kg)	0.45	0.55	0.58	0.59	0.54
RDP^g^ (g/kg)	64.58	51.52	57.97	276.75	125.21

a DM, dry matter.

b CP, crude protein.

c OM, organic matter.

d NDF, neutral detergent fiber.

e ADF, acid detergent fiber.

f FOM, fermented organic matter (kg/kg) = OM(%) × ERDOM (the effective degradation of OM in feedstuff in the rumen, %) × 1 (Kg) / 1 (Kg). ERDOM were obtained from previous research findings (20).

g RDP, rumen-degradable protein (g/kg) = (CP (%) × ERDP (the effective degradation of protein in feedstuff in the rumen, %) × 1 (Kg) × 1,000) / 1 (kg). ERDP values were obtained from previous research findings (20).

**Table 2:** Ingredients and nutrient composition of experimental diets (DM basis%).

Items	Diet (RDP^a^, g/kg)					
	HP-1 (88.90)	HP-2 (83.53)	MP-1 (78.83)	MP-2 (72.17)	LP-1 (67.31)	LP-2 (60.85)
Ingredients (% DM^b^)						
Corn stalk silage	38.66	38.71	38.75	38.80	38.83	38.88
Peanut vine	31.02	31.06	31.09	31.13	31.16	31.20
Corn	10.99	13.36	15.43	17.95	19.88	22.41
Wheat bran	3.90	3.90	3.91	4.54	4.86	5.34
Soybean meals	13.26	10.80	8.65	5.41	3.10	0.00
CaHPO_4_	0.51	0.51	0.51	0.51	0.51	0.51
NaHCO_3_	0.39	0.39	0.39	0.39	0.39	0.39
NaCl	0.25	0.25	0.25	0.25	0.25	0.25
Limestone	0.52	0.52	0.52	0.52	0.52	0.52
Antifungal agent	0.16	0.16	0.16	0.16	0.16	0.16
Premix^c^	0.34	0.34	0.34	0.34	0.34	0.34
Nutrient composition^d^ (DM)						
NE_L_^e^ (MJ/kg)	6.28	6.25	6.22	6.17	6.14	6.09
FOM^f^ (kg/kg)	0.51	0.51	0.51	0.51	0.51	0.51
RDP (g/kg)	88.90	83.53	78.83	72.17	67.31	60.85
RUP (g/kg)	66.6	63.27	59.57	56.43	52.89	49.35
CP (%)	15.55	14.68	13.84	12.86	12.02	11.02
NDF (%)	50.03	49.68	49.37	49.10	48.86	48.56
ADF (%)	30.87	30.92	30.96	31.04	31.09	31.16
Ca (g/kg)	1.46	1.45	1.45	1.44	1.44	1.43
P (g/kg)	0.35	0.35	0.34	0.33	0.33	0.32
DCP^g^ balance (g/d)						
Required^h^	351.21	346.33	353.41	353.75	350.28	350.05
Supplied^i^	619.35	578.93	543.57	492.14	455.05	405.74
Balance	268.14	232.6	190.16	138.39	104.77	55.69

a RDP, dietary rumen-degradable protein (g/kg) were obtained using the equations established by Tang et al. (19). HP-1 and HP-2 represent high RDP; MP-1 and MP-2, medium RDP; and LP-1 and LP-2, low RDP.

b DM, dry matter.

c The premix provided the following per kg of the premix: VA 100,000 IU, VD_3_ 50,000 IU, VE 3,500 IU, Cu 26 mg, Mn 14 mg, Fe 60 mg, Zn 70 mg, I 60 mg, Co 45 mg.

d Nutrient composition was measured, except NE_L_ and DCP.

e NE_L,_ net energy for lactation. NE_L_ values were estimated based on the Chinese Feeding Standard of Dairy Cattle (NY/T 34-2004).

f FOM, dietary fermented organic matter (kg/kg), was obtained using the equations established by Tang et al. (19).

g DCP, Digestible crude protein.

h Required, Required level of DCP was calculated based on the research results of Ruan et al. (22).

i Supplied, Supplied level of DCP was estimated based on the Chinese Feeding Standard of Dairy Cattle (NY/T 34-2004).

### 2.2 Measurements, sampling, and analyses

#### 2.2.1 Determination of growth performance

All experimental buffaloes were fasted for 24 h and weighed at 08:30 h at the beginning and end of the formal experiment to determine the initial body weight (IBW, kg) and final body weight (FBW, kg) and average daily gain (ADG, kg/d) was calculated using the following formula: ADG (kg/d) = (FBW–IBW)/experimental period (53 days) (23). The 53-day period excludes the final 5 days of the formal experiment, during which digestion and metabolism of N were evaluated. The body weight of each buffalo was measured by weighbridge (Model XK3190-E10, Shanghai Yaohua Weighing System Co., Ltd., Shanghai, China). During the data collection phase, the daily feed supply and refusals for each buffalo were recorded weekly. Each buffalo was housed in individual pen (7 m^2^). DMI (kg/d) was computed daily as the difference between feed offered and feed refused. The feed-to-gain ratio (F/G) of each buffalo was calculated based on the mean ADG (kg/d) and mean DMI (kg/d).

#### 2.2.2 Digestive and metabolic profiles of nitrogen

##### 2.2.2.1 Collection of feces and urine samples

Feces and urine samples were collected daily from each buffalo for five consecutive days using 24-h collection periods. Feces and urine samples were collected from dairy buffalo heifers (*n* = 3/treatment) according to the method reported by Tang et al. (19). From day 69 to day 73, the total feces and urine excretion of each buffalo was weighed daily. For each buffalo, 200 g of fresh feces was mixed with 20 mL of a 10% sulfuric acid solution for nitrogen fixation. Fecal samples were then dehydrated in a forced-air oven at 65 °C for 72 h, ground to a powder, and passed through a 1-mm Wiley mill screen (Arthur H. Thomas Co., Philadelphia, PA, United States). These samples were subsequently analyzed for concentrations of DM and CP. From each buffalo, 500 mL of urine was collected and stored at −20 °C for nitrogen content analysis.

##### 2.2.2.2 Collection of feed samples

During the data collection phase, corn silage, peanut vines, and six types of concentrates were sampled weekly. During the digestion and metabolism phases, daily feed intake and refusals were recorded for each buffalo. Concurrently, daily samples of the residual feed were collected. The samples were dried in a forced-air oven at 65 °C for 72 h, ground, and passed through a 1-mm screen using a Wiley mill screen (Arthur H. Thomas Co., Philadelphia, PA, United States). The samples were then analyzed for DM, NDF, ADF, CP, ash, calcium (Ca), and phosphorus (P).

#### 2.2.3 Chemical analyses

Samples were analyzed for DM (method 930.15), CP (method 984.13), and ash (method 942.05) according to the standard procedures of the Association of Official Agricultural Chemists (24). The contents of NDF and ADF were calculated following Van Soest’s procedures (25). The content of P was measured based on the colorimetric nitro-vanado-molybdate method, and the content of Ca was measured using flame spectrophotometry.

#### 2.2.4 Serum biochemistry indices determination

On the final day of the experiment, blood samples (10 mL) were collected from the jugular vein of each buffalo before feeding, centrifuged at 4,000 × g for 15 min at 25 °C using a TDZ5-WS centrifuge (Xiangtan Xiangyi Instrument Co., Ltd., Xiangtan, China), and then the serum was aliquoted into 1.5-mL microtubes and stored at −20 °C for further analysis. The serum contents of total protein (TP), albumin (ALB), globulin (GLOB), alanine aminotransferase (ALT), aspartate aminotransferase (AST), urea, triglyceride (TG), and glucose (GLU) were measured using an automated biochemistry analyzer (Hitachi 7020; Hitachi Co, Tokyo, Japan). Insulin (INS) level was measured using the MAGLUMI 4000 chemiluminescence analyzer (Shenzhen new industries biomedical engineering Co., Ltd., Shenzhen, China).

#### 2.2.5 Rumen sampling and rumen fermentation index analysis

At the end of the trial, ruminal fluid samples were collected from dairy buffalo heifers (*n* = 4/treatment) before the morning feeding, following the methods detailed in a previous study (26). To minimize saliva contamination, the first 50 mL of rumen fluid was discarded. Approximately 200 mL of rumen fluid was then obtained, filtered through a four-layer cheesecloth, and pH was measured utilizing a portable pH meter (PHB-4, INESA Scientific Instrument Co., Ltd., Shanghai, China). The ruminal fluid samples were immediately flash-frozen in liquid nitrogen and stored at −80 °C for further analysis. The concentration of NH3-N in the rumen liquid was measured according to the alkaline phenol-hypochlorite method using a spectrophotometer (T9S, Beijing Purkinje General Instrument Co., Ltd., Beijing, China) (27). The volatile fatty acid (VFA) contents (acetate, butyrate, propionate, isobutyrate, and isovalerate) were measured utilizing a gas chromatograph (Agilent 7890 A, Agilent Technologies, Santa Clara, CA, United States), that was equipped with a an HP-INNOwax column (Agilent, Technologies; 0.32-mm diameter, 0.50-μm film thickness, 30 m long) and a flame ionization detector. Detailed methods are described by Zhao et al. (28).

#### 2.2.6 High-throughput sequencing and bioinformatics analysis

Microbial DNA was extracted from ruminal fluid samples using the DNA Kit (Omega Bio-tek, Norcross, GA, United States) according to manufacturer’s protocols. The extracted microbial DNA was sent to Shanghai Biozeron Biotechnology Co. Ltd. (Shanghai, China) for sequencing of the 16S RNA and ITS1 genes. For 16S rRNA sequencing, the V3–V4 region of the bacterial 16S rRNA gene was amplified by PCR using the 338-F (5′-ACTCCTACGGGAGGCAGCAG-3′) and 806-R (5′-GGACTACHVGGGTWTCTAAT-3′) universal primers. Fungal ITS amplicon sequencing was conducted with the universal primers 1737-F (5′-GGAAGTAAAAGTCGTAACAAGG-3′) and 2043-R (5′-GCTGCGTTCTTCATCGATGC-3′). The PCR products were purified using the AxyPrep DNA Gel Extraction Kit (Axygen Biosciences, United States) and quantified with the QuantiFluor-ST (Promega, United States), following the manufacturers’ protocols. Finally, the Illumina MiSeq platform (Illumina, San Diego, United States) was used for pair-end 2 × 250-bp sequencing. Raw data have been deposited into the NCBI Sequence Read Archive (SRA) database [Accession No. PRJNA1433064].

The raw sequence reads were demultiplexed and quality-filtered utilizing Trimmomatic and subsequently merged with FLASH. Rarefaction analysis was performed using Mothur v.1.21.1 to calculate various diversity indices, such as Chao1, ACE, Simpson, and Shannon (29, 30). The beta diversity analysis was conducted using Euclidean distance matrix to principal component analysis (PCA) (31). For taxonomic classification, the representative 16S rRNA gene sequences were analyzed with the RDP Classifier algorithm^1^ against the SILVA 128 database at a 70% confidence threshold. The ITS sequences were assigned taxonomy using the UNITE 7.0 database.^2^

#### 2.2.7 Calculation of dietary RDP and FOM

The RDP and FOM contents in the diets were determined based on the actual measured values of the dietary components, using the equations established by Tang et al. (19).

#### 2.2.8 Statistical analyses

Statistical analyses were performed using SPSS version 22.0 (IBM Corp., Armonk, NY, United States). The growth performance, ruminal and serum parameters, and the associated rumen microbiome of dairy buffalo heifers were analyzed by a one-way analysis of variance (ANOVA) and Duncan’s multiple comparison test. Differences groups were identified using Tukey’s honest significant difference (HSD) test. The results were considered significant at *p* < 0.05. Orthogonal polynomials were used to assess the linear and quadratic responses of the dependent variables to dietary RDP as the independent variable. Graphing was performed using RStudio software (4.0.3). Principal coordinate analysis (PCA) was conducted using the Vegan package. All remaining figures were generated with the ggplot2 package.

## 3 Results

### 3.1 Growth performance

**Figure 1:**
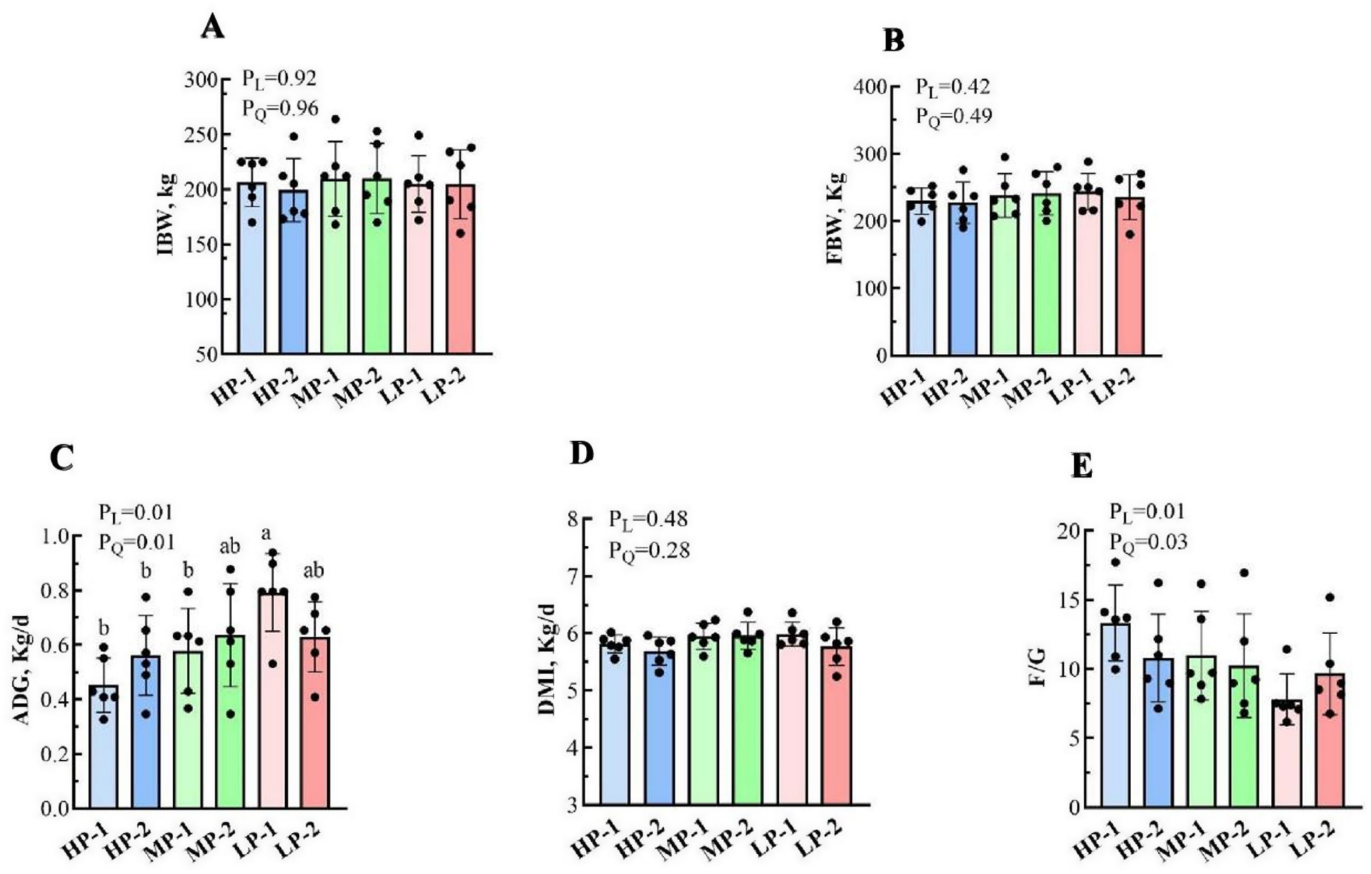
Effects of dietary RDP on growth performance of dairy buffalo heifers. RDP, dietary rumen-degradable protein; HP-1 (dietary RDP, 88.90 g/kg) and HP-2 (dietary RDP, 83.53 g/kg), high RDP; MP-1 (dietary RDP, 78.83 g/kg) and MP-2 (dietary RDP, 72.17 g/kg), medium RDP; LP-1 (dietary RDP, 67.31 g/kg) and LP-2 (dietary RDP, 60.85 g/kg), low RDP. Different letters (a–b) indicate statistically significant difference (*p* < 0.05). *p*-value, probability of a standard, linear or quadratic effect of RDP; IBW, initial body weight; FBW, final body weight; ADG, average daily gain; DMI, dry matter intake; F/G, feed to gain ratio, the ratio of DMI to ADG. **(A)** Initial body weight (IBW); **(B)** Final body weight (FBW); **(C)** Average daily gain (ADG); **(D)** Dry matter intake (DMI); **(E)** Feed to gain ratio (F/G).

The growth performance data of dairy buffalo heifers are presented in Figure 1. The IBW and FBW did not differ among all treatment groups (*p* > 0.05) and ranged from 199.33 to 210 kg and 226.83 to 243.8 kg, respectively. Additionally, dietary RDP levels had no effect on the DMI of buffalo heifers (*p* > 0.05), which varied from 5.77 to 5.99 kg/d across all treatments. The ADG increased linearly and quadratically as dietary RDP levels decreased (*p* < 0.05). Among them, the LP-1 group displayed the highest ADG (0.79 kg/d), which was substantially greater as compared to the HP-1, HP-2, and MP-1 groups (*p* < 0.05), but similar to that of the MP-2 and LP-2 groups (*p* > 0.05). Moreover, the feed conversion ratio (F/G) decreased linearly and quadratically with the reduction of dietary RDP levels (*p* < 0.05). Although no statistically significant differences were observed among treatments (*p* > 0.05), the LP-1 group had the lowest F/G (7.88), suggesting a potential improvement in feed efficiency at this RDP level.

### 3.2 Nitrogen profiles

**Figure 2:**
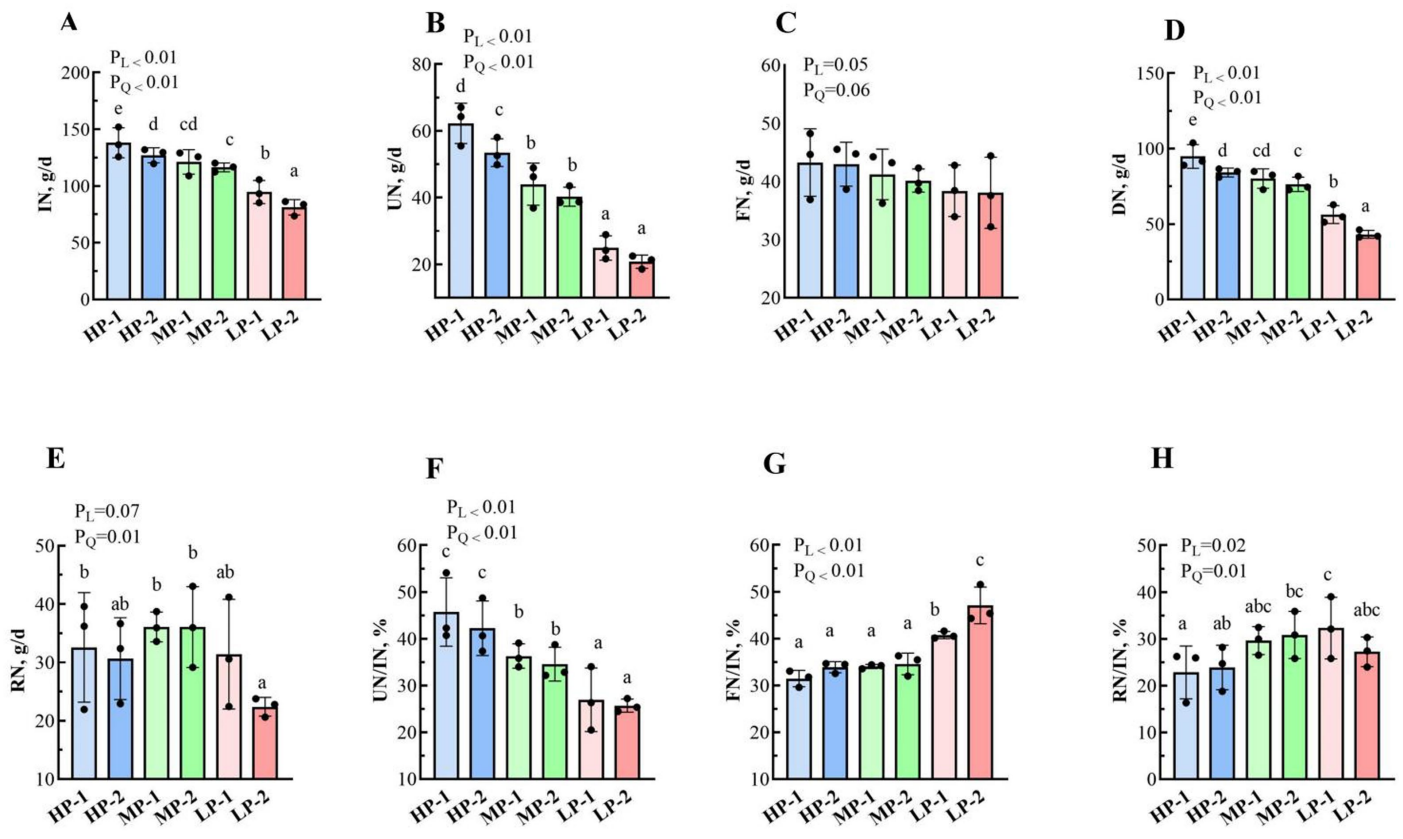
Effects of dietary RDP on the N intake and profiles of dairy buffalo heifers. RDP, Dietary rumen-degradable protein; HP-1 (Dietary RDP, 88.90 g/kg) and HP-2 (Dietary RDP, 83.53 g/kg), high RDP; MP-1 (Dietary RDP, 78.83 g/kg) and MP-2 (Dietary RDP, 72.17 g/kg), medium RDP; LP-1 (Dietary RDP, 67.31 g/kg) and LP-2 (Dietary RDP, 60.85 g/kg), low RDP. Different letters (a–b) indicate statistically significant difference (*p* < 0.05). *p*-value, probability of a standard, linear or quadratic effect of RDP; IN, Intake nitrogen (g/d) = DM intake of diet (g/d) × N content in diet (% DM); UN, Urine nitrogen (g/d) = Urine yield (kg DM/d) × N content in urine (%); FN, Fecal nitrogen (g/d) = Fecal yield (kg DM/d) × N content in fecal (%DM); DN, Digested nitrogen (g/d) = Total N intake (g/d) – Fecal N (g/d); RN, Retention nitrogen (g/d) = Total N intake (g/d) – (Fecal N (g/d) + Urine N (g/d)). **(A)** Intake nitrogen (IN, g/d); **(B)** Urine nitrogen (UN, g/d); **(C)** Fecal nitrogen (FN, g/d); **(D)** Digested nitrogen (DN, g/d); **(E)** Retention nitrogen (RN, g/d); **(F)** UN/IN (%); **(G)** FN/IN (%); **(H)** RN/IN (%).

The N intake and profiles of dairy buffalo heifers are presented in Figure 2. A linear and quadratic decrease (*p* < 0.05) was found in the proportions of intake nitrogen (IN), urinary nitrogen (UN), digested nitrogen (DN), and UN /IN efficiency as dietary RDP decreased. Fecal nitrogen (FN) was not influenced by the dietary RDP (*p* > 0.05). However, the FN/IN efficiency increased linearly and in a quadratic manner with decreasing dietary RDP (*p* < 0.05). The values in the LP-1 and LP-2 groups were significantly higher than those in the HP-1, HP-2, MP-1 and MP-2 groups. The RN and RN/IN efficiency displayed a quadratic elevation with decreasing dietary RDP levels (*p* < 0.05). The RN/IN efficiency (*p* < 0.05) in the LP-1 group (32.31%) was significantly higher than those of the HP-1 group (22.83%) and the HP-2 group (23.87%).

### 3.3 Blood biochemical indices

**Figure 3:**
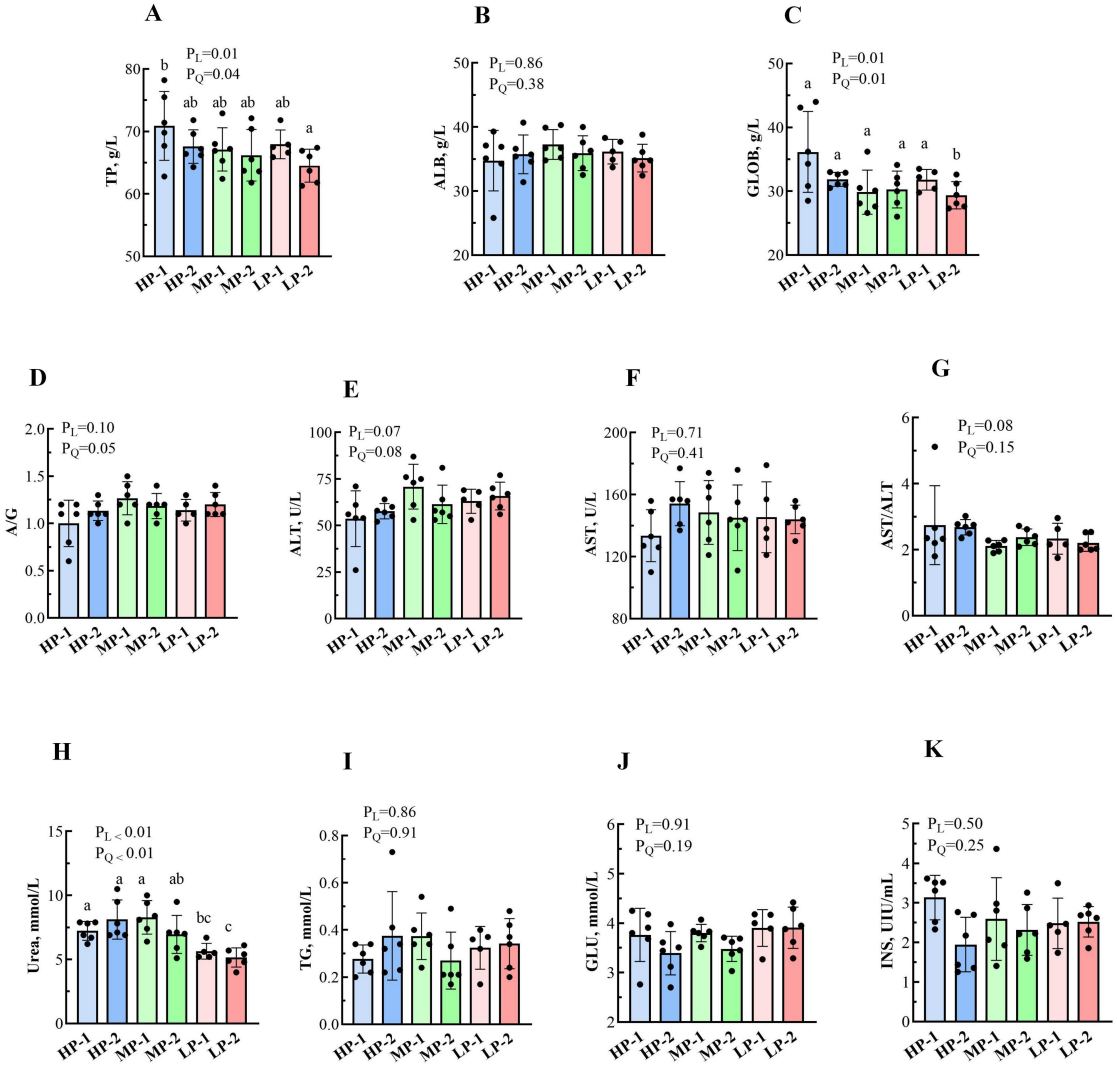
Effects of dietary RDP on the blood biochemical indices of dairy buffalo heifers. RDP, dietary rumen-degradable protein, HP-1 (dietary RDP, 88.90 g/kg) and HP-2 (dietary RDP, 83.53 g/kg), high RDP; MP-1 (dietary RDP, 78.83 g/kg) and MP-2 (dietary RDP, 72.17 g/kg), medium RDP; LP-1 (dietary RDP, 67.31 g/kg) and LP-2 (dietary RDP, 60.85 g/kg), low RDP. Different letters (a–b) indicate statistically significant difference (*p* < 0.05). *p*-value, probability of a standard, linear or quadratic effect of RDP; TP, total protein; ALB, albumin; GLOB, globulin; ALT, alanine aminotransferase; AST, aspartate aminotransferase; TG, triglyceride; GLU, glucose; INS, insulin. **(A)** Total protein (TP); **(B)** Albumin (ALB); **(C)** Globulin (GLOB); **(D)** Albumin to Globulin ratio (A/G); **(E)** Alanine aminotransferase (ALT); **(F)** Aspartate aminotransferase (AST); **(G)** AST/ALT ratio; **(H)** Lactate dehydrogenase (LDH); **(I)** Triglyceride (TG); **(J)** Glucose (GLU); **(K)** Insulin (INS).

Dietary RDP levels influenced several blood biochemical indices (Figure 3). Total protein (TP) concentration showed linear and quadratic effects (*p* > 0.05), whereas albumin concentration showed no substantial difference among treatments (*p* > 0.05). The globulin levels were affected by dietary RDP levels (*p* < 0.05), with the highest value in the HP-1 group (36.15 g/L) and the lowest in the LP-2 group (29.37 g/L). The albumin-to-globulin ratio (A/G) were not different among groups (*p* > 0.05). Among liver function indicators, alanine aminotransferase (ALT), aspartate aminotransferase (AST), and the AST/ALT ratio were not affected by dietary RDP levels (*p* > 0.05). Serum urea concentration was influenced by RDP levels (*p* < 0.05), with the highest concentration in the MP-1 group (8.28 mmol/L) and the lowest in the LP-2 group (5.15 mmol/L). Additionally, energy metabolism indicators, such as triglycerides, glucose, and insulin, were not affected by dietary RDP levels (*p* > 0.05).

### 3.4 Rumen fermentation parameters

**Figure 4:**
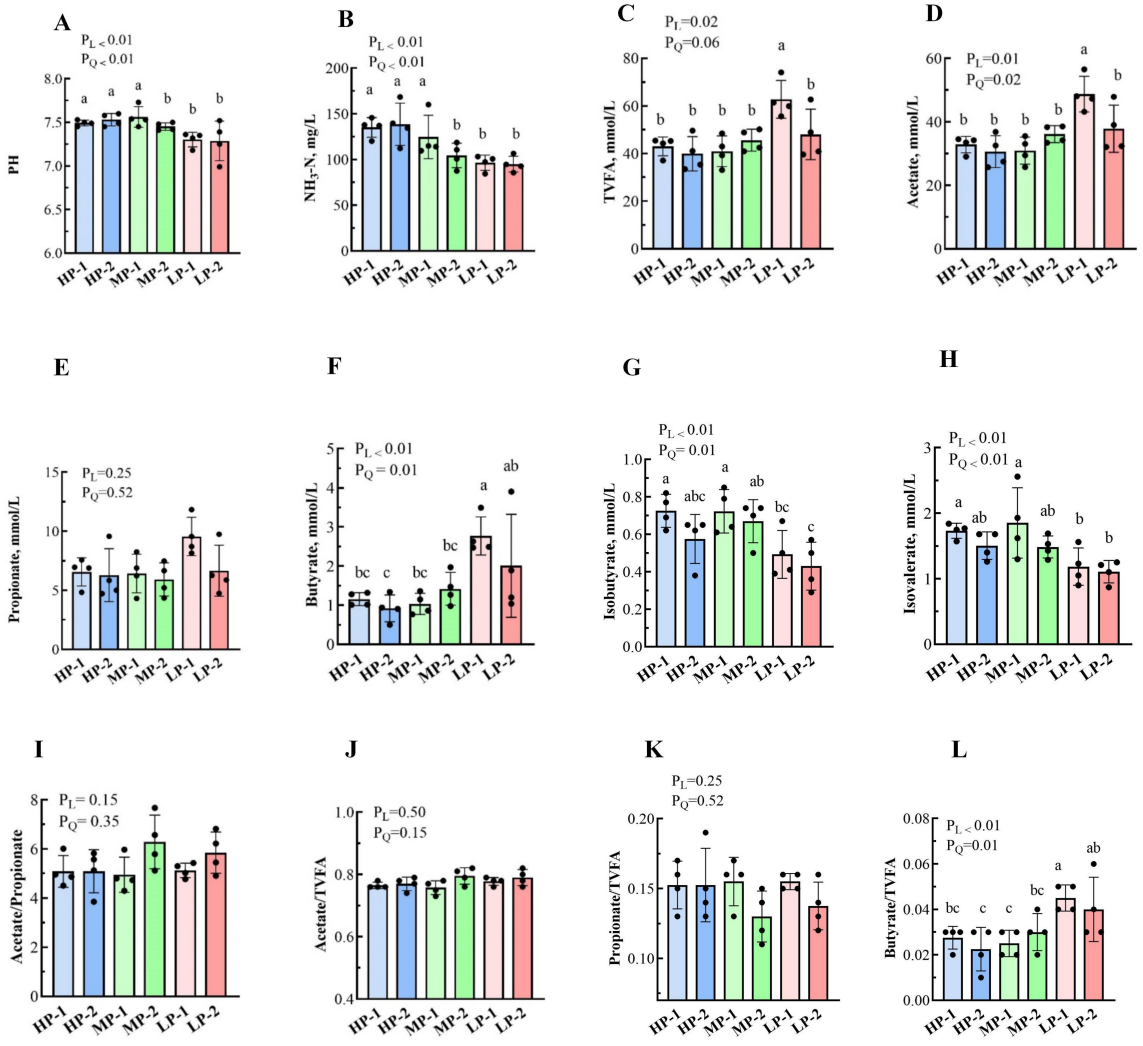
Effects of dietary RDP on the rumen fermentation parameters of dairy buffalo heifers. RDP, dietary rumen-degradable protein, HP-1 (dietary RDP, 88.90 g/kg) and HP-2 (dietary RDP, 83.53 g/kg), high RDP; MP-1 (dietary RDP, 78.83 g/kg) and MP-2 (dietary RDP, 72.17 g/kg), medium RDP; LP-1 (dietary RDP, 67.31 g/kg) and LP-2 (dietary RDP, 60.85 g/kg), low RDP. Different letters (a–b) indicate statistically significant difference (*p* < 0.05). *p*-value, *p*robability of a standard, linear or quadratic effect of RDP; TVFA, total volatile fatty acids. **(A)** pH; **(B)** NH₃-N (mg/L); **(C)** Total volatile fatty acids (TVFA, mmol/L); **(D)** Acetate (mmol/L); **(E)** Propionate (mmol/L); **(F)** Butyrate (mmol/L); **(G)** Isobutyrate (mmol/L); **(H)** Isovalerate (mmol/L); **(I)** Acetate/Propionate ratio; **(J)** Acetate/TVFA (%); **(K)** Propionate/TVFA (%); **(L)** Butyrate/TVFA (%).

Rumen fermentation parameters of dairy buffalo heifers are presented in Figure 4. With the decrease in dietary RDP levels, ruminal pH and NH_3_-N exhibited linear and quadratic decreases (*p* < 0.01), and the values in the HP-1, HP-2, and MP-1 groups were significantly higher than those in the MP-2, LP-1, and LP-2 groups. Alterations in dietary RDP levels yielded significantly higher concentrations of TVFA and acetate in the rumen fluid of the LP-1 group as compared with the other groups (*p* < 0.05), while propionate concentration was not affected (*p* > 0.05). Moreover, butyrate concentration was affected by dietary RDP, with the highest value in the LP-1 group (*p* < 0.05), but no differences in butyrate concentration was found among the LP-1 and LP-2 groups (*p* > 0.05). The concentrations of isobutyrate and isovalerate in rumen fluid also changed substantially (*p* < 0.05), where those in the HP-1 and MP-1 groups were significantly higher than those in the LP-1 and LP-2 groups. The acetate/propionate ratio and the proportions of acetate and propionate in TVFA were not affected by dietary RDP levels (*p* > 0.05). Notably, the butyrate/TVFA ratio in the rumen fluid changed (*p* < 0.05), with the lowest value in the HP-2 group and the highest value in the LP-1 group.

### 3.5 Ruminal microbial community

**Figure 5:**
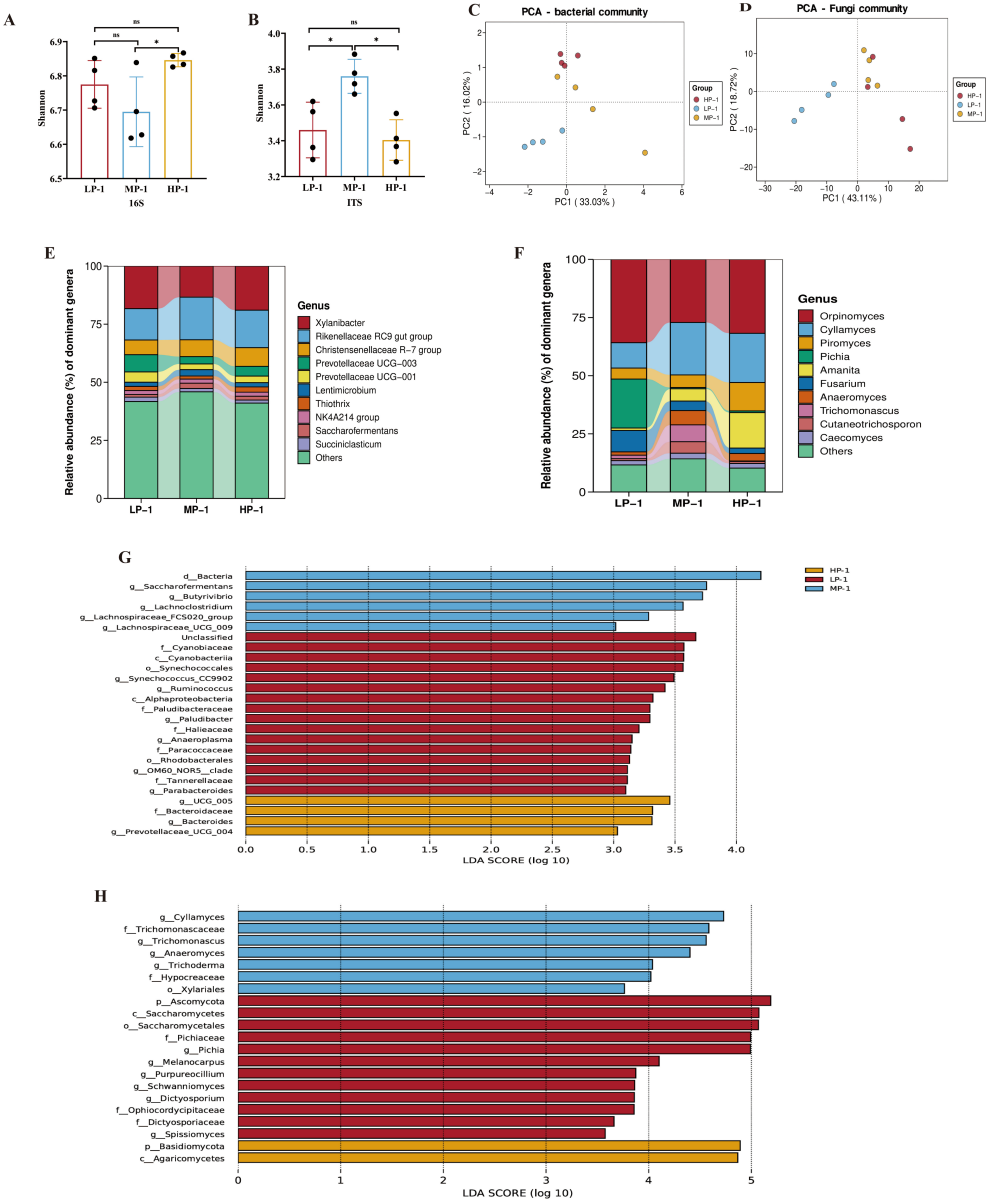
Effects of dietary RDP on the ruminal microbial community of dairy buffalo heifers. RDP, dietary rumen-degradable protein, HP-1 (dietary RDP, 88.90 g/kg) and HP-2 (dietary RDP, 83.53 g/kg), high RDP; MP-1 (dietary RDP, 78.83 g/kg) and MP-2 (dietary RDP, 72.17 g/kg), medium RDP; LP-1 (dietary RDP, 67.31 g/kg) and LP-2 (dietary RDP, 60.85 g/kg), low RDP; **(A)** Shannon for bacterial, **(B)** Shannon for fungi, **(C)** PCA for bacterial, **(D)** PCA for fungi, **(G)** Linear discriminant analysis (LDA) score for bacterial, and **(H)** LDA score for fungi, **(E)** Relative abundance of dominant bacterial genera, **(F)** Relative abundance of dominant fungal genera.

The effects of different dietary RDP levels on the ruminal microbial community are shown in Figure 5. Compared with the MP-1 group, the Shannon index of ruminal bacteria in the HP-1 group was elevated (Figure 5A), while differences in this index between the LP-1 group and the MP-1 or HP-1 groups were not significant. In terms of fungi, the MP-1 group displayed a higher Shannon index than the LP-1 and HP-1 groups (Figure 5B), suggesting that different RDP levels influence the ruminal microbial diversity of buffaloes. Consistent with these results, PCA found that dietary RDP levels significantly influenced the structures of both bacterial and fungal communities (Figures 5C,D).

At the genus level, the relative abundances of dominant bacterial genera (Figure 5E; Supplementary Table S1) and fungal genera (Figure 5F; Supplementary Table S1) varied among different treatments. The five bacterial genera with the highest relative abundances were *Xylanibacter*, *Rikenellaceae RC9 gut group*, *Christensenellaceae R-7 group*, *Prevotellaceae UCG-003*, and *Prevotellaceae UCG-001*. For fungi, the five genera with the highest relative abundances were *Orpinomyces, Cyllamyces*, *Piromyces*, *Pichia*, and *Amanita*. Notably, the relative abundance of *Prevotellaceae UCG-003* and *Prevotellaceae UCG-001* in the LP-1 group showed significantly higher relative abundance compared with those of the HP-1 group and the MP-1 group (*p* < 0.05). Additionally, the relative abundance of *Pichia* in the LP-1 group increased by 28.8-fold and 39.68-fold compared with the HP-1 and MP-1 groups, respectively (*p* < 0.05).

Linear discriminant analysis effect size analysis was conducted to identify marker bacteria and fungi among different groups (Figures 5G,H). *Paludibacter*, *Parabacteroides*, and *Ruminococcus* were the dominant bacteria for the LP-1 group, *Saccharofermentans* and *Butyrivibrio* were the dominant bacteria for the MP-1 group, and *UCG_005* and *Bacteroides* were the dominant bacteria for the HP-1 group. For fungi, *Pichia* and *Melanocarpus* were enriched in the LP-1 group, and *Anaeromyces* was enriched in the MP-1 group (Figure 5H). However, the marker fungi in the HP-1 group could only be identified at the class level, namely *Agaricomycetes*. These results demonstrate RDP-dependent shifts in microbial taxa, which may have implications for ruminal metabolism.

### 3.6 Correlation analysis of the growth phenotype and rumen fluid parameters and major bacteria

**Figure 6:**
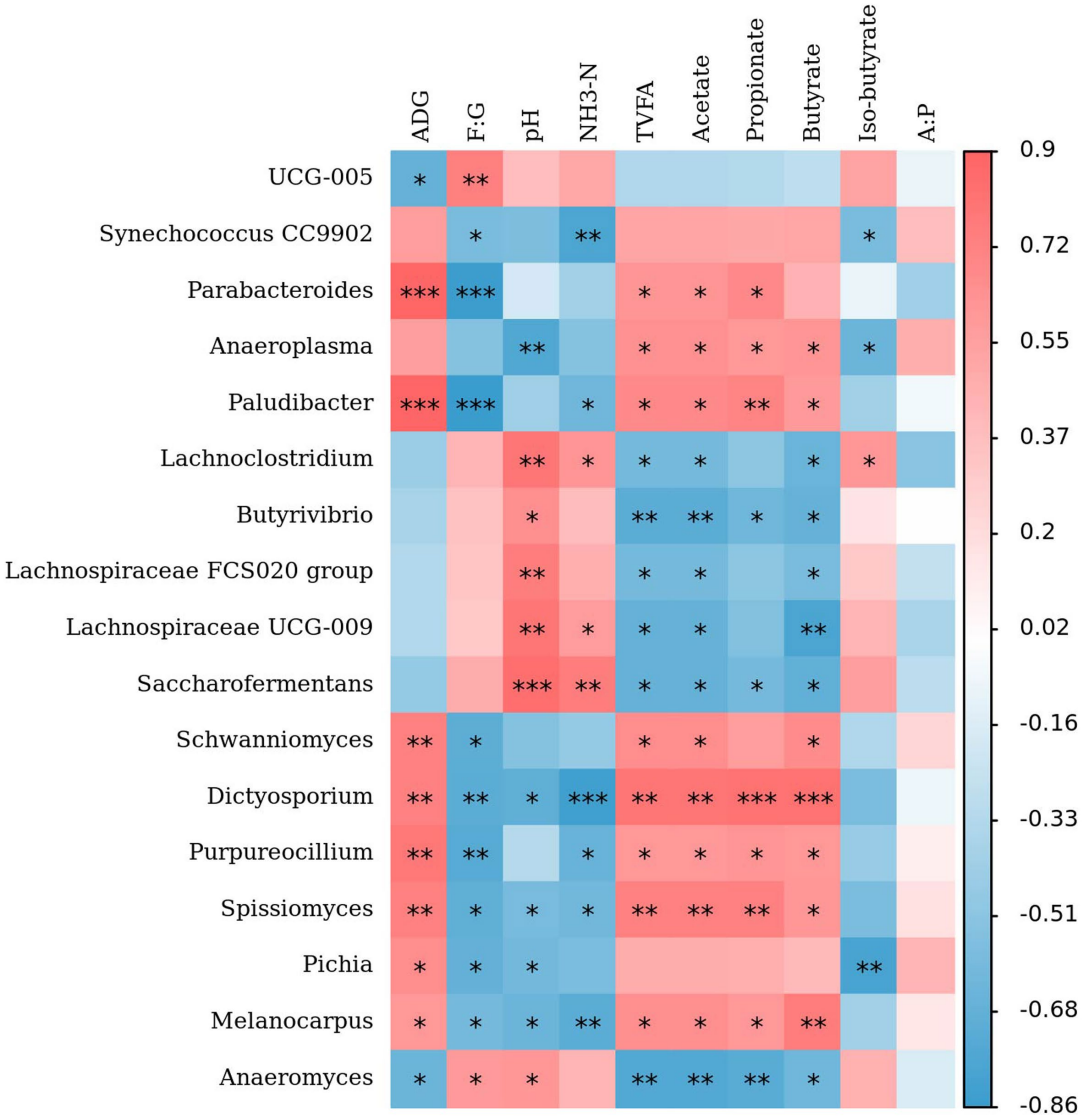
Spearman correlation between the growth phenotype and rumen fluid parameters of buffalo heifer and the bacterial populations. *p < 0.05, **p < 0.01, and ***p < 0.001. ADG, average daily gain; F/G, feed to gain ratio, the ratio of DMI to ADG; A: P, acetate/propionate.

Spearman correlation analysis between signature microbial taxa under different dietary RDP levels and rumen fermentation parameters as well as growth performance revealed that microbes enriched in the LP-1 group (e.g., *Parabacteroides*, *Paludibacter*, *Schwanniomyces*, *Dictyosporium*, *Purpureocillium*, *Spissiomyces*, *Pichia*, and *Melanocarpus*), were positively correlated with ADG and negatively correlated with F/G (Figure 6). In contrast, *UCG-005* and *Anaeromyces* enriched in the HP-1 group displayed the opposite correlation with ADG and F/G compared to those in the LP-1 group. Most microbes in the LP-1 group were negatively correlated with pH and NH_3_-N but positively correlated with VFA concentrations. Conversely, microbes enriched in the MP-1 and HP-1 groups demonstrated opposite correlation trends with pH, NH_3_-N, and VFAs relative to those in the LP-1 group. Specifically, *Parabacteroides*, *Anaeroplasma*, *Paludibacter*, *Schwanniomyces*, *Dictyosporium*, *Purpureocillium*, *Spissiomyces*, and *Melanocarpus* in the LP-1 group were positively correlated with VFAs, while *Anaeromyces*, *Lachnoclostridium*, *Butyrivibrio*, *Lachnospiraceae FCS020 group*, *Lachnospiraceae UCG-009*, and *Saccharofermentans* in the MP-1 and HP-1 groups were positively correlated with VFAs.

## 4 Discussion

### 4.1 Effects of dietary RDP on the growth performance of dairy buffalo heifers

The core goal of ruminant protein nutrition is to optimize rumen microbial protein (MCP) synthesis by supplying an appropriate amount of RDP, thereby achieving the desired animal productivity with the minimum dietary CP input (8, 32, 33). When carbohydrates are sufficiently available, increasing the dietary RDP content promotes MCP synthesis, which in turn, enhances rumen fermentation and improves animal growth performance (8, 9, 34). However, the present study obtained contrasting results. As dietary RDP levels were reduced, the ADG of 7–10-month-old dairy buffalo heifers increased linearly, and the ADG of the high RDP group (HP-1, 88.90 g/kg DM) was 75% lower than that of the low RDP group (LP-1, 67.31 g/kg DM). This finding indicates that, within the range tested, the RDP levels in the LP-1 group may have met the N demand of rumen microbes. Such counterintuitive results are most likely attributed to the imbalance between rumen available N and carbohydrates in the high RDP groups, which inhibited both MCP synthesis and rumen fermentation. Specifically, when RDP exceeds the microbial N requirement, excess NH_3_-N is produced in the rumen. This excess NH_3_-N fails to contribute to MCP synthesis and may suppress the activity of fiber-degrading bacteria (e.g., Ruminococcus and Butyrivibrio). This results in decreased decomposition of structural carbohydrates and the production of VFAs, the primary energy source for ruminants, which accounts for 70–80% of their total energy supply (35–38). Furthermore, MCP is a high-quality source of amino acids (AA), with an AA profile closely matching that of animal body tissues. MCP contributes 82.4% of the total AA supply to ruminants and has a small intestinal digestibility of approximately 80% (37, 38). Thus, the decreased ADG in the high-RDP groups can be further explained by the N-carbon imbalance in the rumen, which inhibited MCP synthesis and reduced the amount of absorbable AA in the small intestine, ultimately limiting body protein deposition and growth.

A gap in previous studies is that most focus on lactating cows or older buffalo heifers, while research on the RDP requirements of 7–10-month-old prepubertal dairy buffalo heifers, a key stage for skeletal and physiological development, remains scarce. For instance, Gressley and Armentano (39) reported that insufficient RDP (7.4% DM) reduced the DMI of lactating cows, and our previous study on 13–15-month-old buffalo heifers also revealed that a higher dietary RDP content (8.04% vs. 6.65% DM) was beneficial for increasing DMI (19). In contrast, the current study demonstrated that a dietary RDP level of 6.73% DM appeared to be sufficient to meet the N demand of rumen microbes, and the RDP levels had no effect on the DMI of 7–10-month-old heifers. These contradictory results may stem from differences in diets used across different batches of experiments (e.g., concentrate ingredients, forage types), as well as differences in animal breeds, physiological stages, and environmental conditions (8).

F/G is a key indicator reflecting the production efficiency of ruminants, as it integrates both DMI and ADG. In this study, the DMI of heifers in the low-RDP groups was comparable to that in the high-RDP groups, but their ADG was substantially higher, ultimately leading to a lower F/G in the LP-1 group (7.88) and thus higher feed efficiency. This result is consistent with the findings of a study on lactating cows, where increasing RDP from 9.5 to 11.7% DM did not improve milk yield, indicating that increasing RDP levels provides no additional benefits when digestible crude protein (DCP) requirements are met. However, the present study confirms this efficiency principle in 7–10-month-old prepubertal buffalo heifers. Specifically, we demonstrated that reducing RDP to 6.73% DM does not compromise DMI; it also enhances ADG, thereby improving feed efficiency. This provides a crucial theoretical basis for formulating cost-effective diets for this specific age group (8, 40).

### 4.2 Effects of dietary RDP on N intake and profiles of dairy buffalo heifers

Inadequate or excessive RDP disrupts nitrogen utilization, impairing rumen microbial function and increasing nitrogen excretion (41, 42). Therefore, optimizing dietary RDP is essential to enhance feed efficiency and mitigate nitrogen emissions. This study observed a linear decrease in IN (*p* < 0.05) with decreasing dietary RDP, which could be the result of a reduced CP concentration in the feed, with no substantial difference in the DMI among groups (*p* > 0.05). In addition, we found that the UN excretion and the UN /IN efficiency decreased linearly (*p* < 0.05) with decreasing dietary RDP level, suggesting that low CP-RDP diets effectively reduced UN output and UN /IN efficiency in dairy buffalo heifers. The observed linear decreases in UN excretion and UN /IN efficiency were expected, as dietary RDP in excess of microbial requirements is degraded into ammonia-N, absorbed, converted to urea in the liver, and subsequently excreted in urine (43). Similarly, the UN and UN /IN efficiency significantly decreased (*p* < 0.05) when the RDP or CP in the diet was decreased in dairy cows (44, 45), growing goat kids (17), and buffaloes (19). A linear decrease (*p* < 0.05) in the DN with declining dietary RDP was also observed, which could be attributed to the corresponding linear decrease (*p* < 0.05) in the proportion of IN, while FN was not affected by decreasing dietary RDP. Similarly, a linear reduction of DN in response to decreasing dietary RDP has been reported in dairy cows (46). RN and RN/IN are significant indicators that reflect nitrogen use efficiency in animals. A quadratic increase (*p* < 0.05) in both the RN and RN/IN efficiency was observed with decreasing dietary RDP. Notably, the RN/IN efficiency was significantly higher (*p* < 0.05) in the LP-1 group (32.31%) as compared to the HP-1 and HP-2 groups (22.83 and 23.87%, respectively), suggesting that the diet of LP-1 group (RDP = 67.31 g/kg, CP = 12.02%) may be beneficial for improving the NUE of dairy buffalo heifers and may be the result of synchronization of energy supply and microbial protein synthesis in the rumen. Diets containing 67.31 g/kg RDP supported maximal ADG in dairy buffalo heifers with minimal N excretion to the environment as compared with diets with higher RDP content. Similarly, Zeleke et al. (47) reported that the nitrogen utilization efficiency of lactating dairy cows was reduced 9.03% (*p* < 0.05) when dietary CP increased from 15 to 17%. Sun et al. (48) also reported a linear decrease (*p* < 0.05) in RN and RN/IN efficiencies with increasing dietary RDP levels in dairy cows. However, RN and RN/IN efficiencies in beef heifers were unaffected by rumen degradable starch and RDP levels (49). These contrasting results could be due to differences between animals and diets.

### 4.3 Effects of dietary RDP on serum biochemistry indices of dairy buffalo heifers

This study confirmed that decreasing dietary RDP (from 88.90 g/kg to 67.31 g/kg DM) did not adversely affect the liver and kidney function or glucose-lipid metabolism indices of the heifers. For example, the concentrations of ALT, AST, TG, and GLU differences among groups were not significant. This suggests that the low-RDP diet did not impose metabolic stress on the heifers. In the present study, the serum TP concentrations were decreased as dietary RDP levels decreased. Our result was consistent with previous results showing that the serum TP and albumin concentration of assaf lambs decrease with declining dietary crude protein levels (50). Furthermore, the linear decrease in serum urea concentration was consistent with the reduction in rumen NH_3_-N concentration across groups—a result that aligns with the classic rumen N metabolism pathway. In contrast, the low-RDP groups in this study displayed lower serum urea and ruminal NH_3_-N concentrations—a pattern that might potentially favor N utilization efficiency. Specifically, a higher proportion of dietary N was allocated to MCP synthesis rather than being excreted primarily as urea. This finding can help reduce feed costs by decreasing dietary CP inclusion levels, It can also help mitigate N pollution, thereby facilitating sustainable development of dairy buffalo production systems.

### 4.4 Effects of dietary RDP on rumen fermentation of dairy buffalo heifers

Rumen fluid parameters, including pH, NH_3_-N, and VFA concentrations, are indicators reflecting rumen fermentation status and health (51). In the present study, low RDP levels increased TVFA concentrations while decreasing rumen fluid pH and NH3-N concentration. This phenomenon suggests that, in this study, rumen nitrogen may have been sufficiently used for microbial protein synthesis, thereby promoting rumen fermentation. Furthermore, the lowest NH3-N concentration in the LP-2 group was 94.91 mg/L, which remained above the minimum requirement for rumen microbial growth (≥50 mg/L), suggesting that the RDP supply was likely adequate in our experiment (52). Furthermore, a previous *in vitro* study reported that increasing dietary CP from 14 to 16% (thereby raising the RDP level from 55 to 65% on a CP basis) reduced nutrient digestibility, microbial protein synthesis (MPS), and VFA concentrations. The underlying mechanism for this decreased fermentation efficiency under high RDP levels is the disruption of protein-energy synchronization, which inhibits rumen microbial activity (53). Notably, VFAs produced by rumen microbial fermentation satisfies up to 70% of the total energy requirement in ruminants (54). Our finding that reducing dietary CP and RDP levels increased TVFA content is consistent with a previous study, which demonstrated that high dietary CP and RDP concentrations decreases the feed protein degradation efficiency, leading to a reduction in the production of VFAs (55).

### 4.5 Effects of dietary RDP on the associated rumen microbiome in dairy buffalo heifers

It is well recognized that dietary composition is a major driver of microbial community shifts (56). However, there is a paucity of research evaluating the effects of dietary RDP levels on the rumen microbiota of water buffaloes, which limits our understanding of how RDP regulates rumen function and production performance in this specific ruminant species (57, 58). The present study addresses this knowledge gap and demonstrates that different dietary RDP levels alter the diversity of bacterial and fungal communities in dairy buffalo heifers. This microbial variation is one of the major factors contributing to the variation in rumen fermentation parameters and production performance observed across groups. Specifically, in the LP-1 group, the rumen dominant bacterial genera such as *Prevotellaceae UCG-003* and *Prevotellaceae UCG-001* showed significantly higher relative abundance compared with those of the HP-1 group and the MP-1 group. Both *Prevotellaceae UCG-003* and *Prevotellaceae UCG-001* belong to bacteria *Prevotella*, which plays a key role in the metabolisms of protein. Certain *Prevotella* strains can produce acetate, succinate, and propionate (59). *Pichia* is a predominant eukaryotic fungal genus in the rumen of ruminants and exhibits high cellulase activity, enabling the degradation of cellulose and hemicellulose into monosaccharides (60). Furthermore, *Pichia* may directly participate in the deamination of amino acids, leading to ammonia production. Consequently, a high biomass of this genus could supply rumen microbiota with essential nutrients—including organic acids, peptides, and amino acids (61), thereby enhancing feed digestibility and animal performance (62). In this study, we found that the relative abundance of *Pichia* in the LP-1 group increased by 28.8-fold and 39.68-fold compared with the HP-1 and MP-1 groups, respectively (*p* < 0.05). In addition, in this study we found that low dietary RDP levels were enriched for *Ruminococcus*, *Parabacteroides*, *Paludibacter*, *Schwanniomyces*, *Dictyosporium, Purpureocillium*, *Spissiomyces*, *Pichia*, and *Melanocarpus.* These genera were positively correlated with ADG, feed efficiency, and rumen fluid VFA concentrations. Notably, *Parabacteroides* and *Paludibacter* showed the strongest positive correlations with ADG. This aligns with previous studies in other ruminants (63), where *Parabacteroides* and *Paludibacter* were reported to be associated with enhanced nutrient utilization and growth performance, likely due to their roles in fiber degradation and microbial protein synthesis.

### 4.6 The limitations of the study

First, although animals of different genetic types (Murrah, Nili-Ravi, Mediterranean hybrid, and triple-crossbred) were evenly and randomly distributed across all dietary treatment groups, the potential influence of genetic background on the measured outcomes cannot be completely excluded. Second, rumen fluid was not collected at the beginning of the experiment; therefore, pre-existing differences in rumen microbiota among groups cannot be ruled out. Third, the sample size of six heifers per treatment is relatively modest.

## 5 Conclusion

This study demonstrates that the optimal dietary RDP content for 7–10-month-old dairy buffalo heifers is 67.31 g/kg (LP-1 group). This level maintains dry matter intake, enhances the ADG and feed efficiency, improves nitrogen utilization and rumen fermentation—all without compromising physiological health. The observed effects were associated with distinct rumen microbial changes, particularly an increased abundance of *Pichia*, a eukaryotic fungal genus recognized for its high cellulolytic activity and its role in nutrient provisioning within rumen ecosystems. Thus, this study provides a basis for precise protein nutrition regulation and green farming of heifers at this stage.

## Data Availability

The original contributions presented in the study are publicly available. Raw data have been deposited into the NCBI Sequence Read Archive (SRA) database (Accession No.PRJNA1433064).

## Footnotes

1
http://rdp.cme.msu.edu/

2
https://unite.ut.ee/
