# Supplementary material for: Effects of dietary rumen-degradable protein on growth performance, nitrogen metabolism, and rumen microbiome in dairy buffalo heifers
Source: Front Vet Sci. 2026 Apr 22;13:1806578. doi: 10.3389/fvets.2026.1806578 (PMC13143584; doi:10.3389/fvets.2026.1806578)
Supplement: Supplementary file 1 [file Table_1.docx]

Supplementary Material

Table S1 Effects of dietary RDP on Relative abundance of rumen the bacterial genus and fungal genera of dairy buffalo heifers

| **Items** | **Diet (RDP^1^, g/kg)** | | | **SEM** | ***P*-value^2^** | | |
| --- | --- | --- | --- | --- | --- | --- | --- |
|  | - **HP-1**   **(88.90)** | - **MP-1**   **(78.83)** | - **LP-1**   **(67.31)** |  | **Standard** | **Linear** | **Quadratic** |
| Relative abundance of the bacterial genus (%) | | | | | | | |
| *Xylanibacter* | 18.97 | 13.38 | 18.34 | 1.56 | 0.30 | 0.88 | 0.30 |
| *Rikenellaceae RC9 gut group* | 16.16 | 18.34 | 13.47 | 1.07 | 0.18 | 0.33 | 0.18 |
| *Christensenellaceae R-7 group* | 8.04 | 7.25 | 6.32 | 0.44 | 0.30 | 0.11 | 0.30 |
| *Prevotellaceae UCG-003* | 4.19^a^ | 3.13^a^ | 7.40^b^ | 0.74 | 0.03 | 0.07 | 0.03 |
| *Prevotellaceae UCG-001* | 2.84^a^ | 2.46^a^ | 4.44^b^ | 0.36 | 0.04 | 0.06 | 0.04 |
| *Lentimicrobium* | 1.80^a^ | 2.74^b^ | 1.78^a^ | 0.18 | 0.02 | 0.97 | 0.02 |
| *Thiothrix* | 2.26 | 1.35 | 1.79 | 0.19 | 0.14 | 0.32 | 0.14 |
| *NK4A214 group* | 1.76 | 1.74 | 1.68 | 0.15 | 0.98 | 0.86 | 0.98 |
| *Saccharofermentans* | 1.63^a^ | 2.26^b^ | 1.19^a^ | 0.17 | 0.01 | 0.30 | 0.01 |
| *Succiniclasticum* | 1.32 | 1.48 | 1.88 | 0.21 | 0.60 | 0.31 | 0.60 |
| Others | 41.04 | 45.88 | 41.71 | 1.32 | 0.29 | 0.85 | 0.29 |
| Relative abundance of the fungal genus (%) | | | | | | | |
| *Orpinomyces* | 31.78 | 27.11 | 35.80 | 2.44 | 0.38 | 0.53 | 0.38 |
| *Cyllamyces* | 21.15 | 22.55 | 10.92 | 2.39 | 0.08 | 0.09 | 0.08 |
| *Piromyces* | 12.24 | 5.42 | 4.71 | 1.47 | 0.06 | 0.03 | 0.05 |
| *Pichia* | 0.73^b^ | 0.53^b^ | 21.03^a^ | 3.40 | <0.01 | <0.01 | <0.01 |
| *Amanita* | 15.22^b^ | 5.30^ab^ | 1.06^a^ | 2.75 | 0.03 | 0.03 | 0.08 |
| *Fusarium* | 2.35 | 4.10 | 9.21 | 1.68 | 0.24 | 0.1 | 0.24 |
| *Anaeromyces* | 3.21^a^ | 6.10^b^ | 1.42^a^ | 0.74 | 0.01 | 0.34 | 0.01 |
| *Trichomonascus* | 0.15 | 7.27 | 1.51 | 1.45 | 0.09 | 0.72 | 0.09 |
| *Cutaneotrichosporon* | 0.89 | 4.94 | 0.86 | 0.83 | 0.06 | 0.99 | 0.05 |
| *Caecomyces* | 2.01 | 2.43 | 1.85 | 0.16 | 0.33 | 071 | 0.33 |
| Others | 10.28 | 14.25 | 11.63 | 1.15 | 0.39 | 0.65 | 0.39 |

^1^RDP, dietary rumen-degradable protein; HP-1 and HP-2, high RDP; MP-1 and MP-2, medium RDP; LP-1 and LP-2, low RDP.^a–b^Values with different superscripts within each row are significantly different (*P* < 0.05).^2^P-value, probability of a standard, linear or quadratic effect of RDP.
